# Zeolite addition to improve biohydrogen production from dark fermentation of C5/C6-sugars and *Sargassum* sp. biomass

**DOI:** 10.1038/s41598-021-95615-1

**Published:** 2021-08-11

**Authors:** R. M. Silva, A. A. Abreu, A. F. Salvador, M. M. Alves, I. C. Neves, M. A. Pereira

**Affiliations:** 1 CEB-Centre of Biological Engineering, University of Minho, Campus de Gualtar, 4710-057 Braga, Portugal; 2 CQUM-Centre of Chemistry, University of Minho, 4710-057 Braga, Portugal

**Keywords:** Environmental biotechnology, Environmental microbiology

## Abstract

Thermophilic biohydrogen production by dark fermentation from a mixture (1:1) of C5 (arabinose) and C6 (glucose) sugars, present in lignocellulosic hydrolysates, and from *Sargassum* sp*.* biomass, is studied in this work in batch assays and also in a continuous reactor experiment. Pursuing the interest of studying interactions between inorganic materials (adsorbents, conductive and others) and anaerobic bacteria, the biological processes were amended with variable amounts of a zeolite type-13X in the range of zeolite/inoculum (in VS) ratios (Z/I) of 0.065–0.26 g g^−1^. In the batch assays, the presence of the zeolite was beneficial to increase the hydrogen titer by 15–21% with C5 and C6-sugars as compared to the control, and an increase of 27% was observed in the batch fermentation of *Sargassum* sp. Hydrogen yields also increased by 10–26% with sugars in the presence of the zeolite. The rate of hydrogen production increased linearly with the Z/I ratios in the experiments with C5 and C6-sugars. In the batch assay with *Sargassum* sp., there was an optimum value of Z/I of 0.13 g g^−1^ where the H_2_ production rate observed was the highest, although all values were in a narrow range between 3.21 and 4.19 mmol L^−1^ day^−1^. The positive effect of the zeolite was also observed in a continuous high-rate reactor fed with C5 and C6-sugars. The increase of the organic loading rate (OLR) from 8.8 to 17.6 kg m^−3^ day^−1^ of COD led to lower hydrogen production rates but, upon zeolite addition (0.26 g g^−1^ VS inoculum), the hydrogen production increased significantly from 143 to 413 mL L^−1^ day^−1^. Interestingly, the presence of zeolite in the continuous operation had a remarkable impact in the microbial community and in the profile of fermentation products. The effect of zeolite could be related to several properties, including the porous structure and the associated surface area available for bacterial adhesion, potential release of trace elements, ion-exchanger capacity or ability to adsorb different compounds (i.e. protons). The observations opens novel perspectives and will stimulate further research not only in biohydrogen production, but broadly in the field of interactions between bacteria and inorganic materials.

## Introduction

Hydrogen is a good energy carrier and an alternative to fossil fuels since it can be produced from renewable sources, it has a high efficiency of conversion to usable power, and non-polluting nature^1^. Dark fermentation is a biological process where hydrogen and carbon dioxide are produced by anaerobic microorganisms in one step^2^. This process is environmentally friendly and one of the most promising and sustainable processes for hydrogen production. Indeed, dark fermentation can be applied for the valorisation of organic wastes such as marine biomass^3^. *Sargassum* sp. is a genus of brown macroalgae that has a global occurrence and it is considered a marine waste. This macroalgae is composed by easily hydrolysable sugars and proteins and has low fractions of lignin and high fractions of hemicellulose as well as a good hydrolysis yield^4^. Due to its characteristics, *Sargassum* sp. can be a suitable candidate for biohydrogen production, turning dark fermentation more competitive and economically sustainable^3^.

Although dark fermentation is a promising process, the production of large quantities of soluble metabolic products, results in low hydrogen production efficiencies and low hydrogen yields (around 25%)^5^. Nevertheless, biological processes efficiencies under anaerobic conditions could be improved by the addition of different types of materials^6^. The mechanism of action of these materials depends on several properties such as electric conductivity or surface area. Zeolites is an aluminosilicate material that have been extensively studied in different biological processes due to its properties, i.e., ion exchange capacity, high surface area and porosity^7^. Several studies reported that the addition of zeolites can lead to a better performance of the anaerobic digestion process (AD) both under mesophilic and thermophilic conditions^8,9^. In these biological processes, zeolites were acting either as ion-exchangers for the removal of ammonia and/or as free ammonia adsorbing material, avoiding the ammonia inhibitory effect towards the microbial community, and therefore improving the overall process^8,9^. Compounds other than ammonia, i.e., cations such as Ca^2+^ and Mg^2+^ and long chain fatty acids, were also reported to be adsorbed to zeolites thus reducing their availability and potential toxicity, which resulted in significant improvements in methane production^10,11^. Moreover, zeolites induced changes in anaerobic microbial communities that apparently promoted the growth of a more efficient methanogenic community^12^.

Regarding the effect of zeolites on biohydrogen production, some studies reported enhancement of hydrogen yields during a two-step process of dark- and photo-fermentation after zeolites addition^13,14^. Again, the beneficial effect of zeolites was linked to its ion-exchanger property and the reduction of ammonium concentration. The hydrogen yield was enhanced in approximately 70% after ammonium removal by zeolites from dark fermentation residual solution^13,14^. Zeolites are also be used as adsorbents in industrial wastewaters for example to remove heavy metals^15,16^. In the majority of the studies, zeolites effect on the biological process of AD or photo-fermentation was assigned to the removal of ammonia or other compounds from the system. However, the high surface area of zeolites and its porosity are another important feature of zeolites for biological processes^7^. These characteristics allow this material to be used as a support for the immobilization of microorganisms in different types of bioreactor configurations improving its performance^7,8^. Recently, it was reported the enhancement of biohydrogen production in a hybrid bioreactor with integrated chlorinated polyethylene (CPE) fixed-bed and with natural and Fe-modified zeolite as a microorganism nutrition carrier (MNC)^17^. The authors observed the enhancement on biohydrogen production during AD process with the Fe-modified zeolite^17^. The authors suggested that Fe-modified zeolite coupled with the CPE fixed-bed could function not only for microbial immobilization but also could induced favourable pathways and enzymes, micronutrient supplementation and electrical conductivity when compared with natural zeolite^17^. Since zeolites successfully improved the efficiency of several biological processes, it was hypothesized that a commercially available zeolite could also improve biohydrogen production by dark fermentation. Therefore, in this study, the effect of zeolite (type-13X) in biohydrogen production by dark fermentation of a mixture (1:1) of C5 (arabinose) and C6 (glucose) sugars present in lignocellulosic hydrolysates and of *Sargassum* sp. biomass, was investigated under thermophilic conditions (70 ± 2 °C).

## Results

### Effect of zeolite on biohydrogen production by dark fermentation

**Figure 1:**
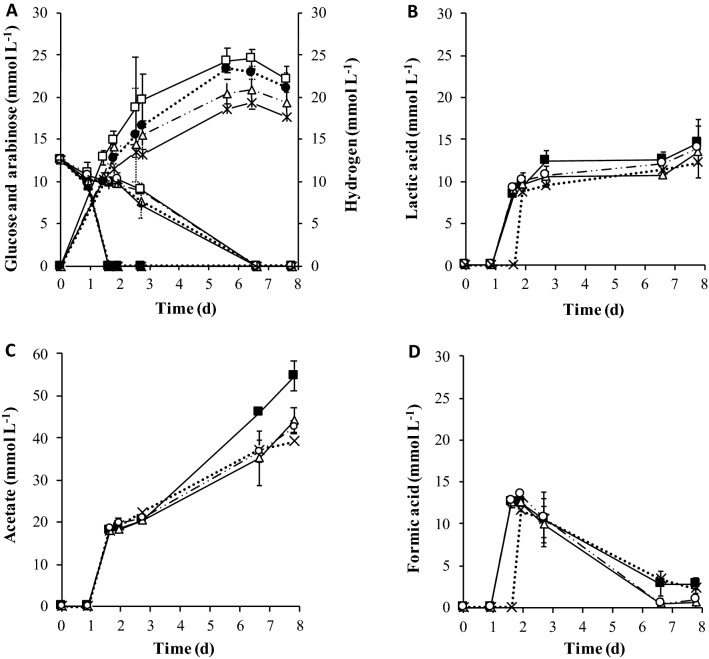
Cumulative hydrogen production and fermentative products produced in the batch assay with different zeolite to inoculum (in VS) ratios (0.26, 0.13, 0.065 g g^−1^) and for the control (without zeolite). (**A**) Glucose consumption:  0.26 g g^−1^;  0.13 g g^−1^;  0.065 g g^−1^;  control. Arabinose consumption:  0.26 g g^−1^;  0.13 g g^−1^;  0.065 g g^−1^;  control. Hydrogen production:  0.26 g g^−1^;  0.13 g g^−1^;  0.065 g g^−1^;  control. (**B**), (**C**) and (**D**) Lactic acid, Acetic acid and formic acid production, respectively:  0.26 g g^−1^;  0.13 g g^−1^;  0.065 g g^−1^;  control. The results are the average and standard deviations for duplicate assays.

The effect of zeolite on biohydrogen production from a mixture of C5 and C6-sugars was evaluated in batch experiments. The presence of zeolite type-13X influenced positively the maximum hydrogen production, the hydrogen yield, and the initial rate of hydrogen production (Fig. 1, Table 1). There was no correlation between the mass of zeolite/mass of inoculum (in VS) ratio (Z/I) and the maximum production or the hydrogen yield. The highest hydrogen production (24.54 mmol L^−1^), was achieved for the Z/I of 0.26 g g^−1^ followed by 22.88 mmol L^−1^ obtained for the Z/I of 0.065 g g^−1^, which were respectively 21% and 15% higher than the obtained for the control assay without zeolite (19.36 mmol L^−1^) (*p* < 0.05) (Fig. 1 A). Yet, no significant differences for hydrogen production between Z/I of 0.26 and 0.065 g g^−1^ were observed (*p* > 0.05). For the Z/I of 0.13 g g^−1^ of inoculum (in VS) hydrogen production reached 20.88 mmol L^−1^, which corresponded to an increment of only 7% compared to the control without zeolite (*p* > 0.05).

**Table 1:** Hydrogen yields, pH and COD balance from batch experiments with C5 and C6-sugars.

Substrate	Z/I (g g^−1^)	Hydrogen yield^a^ (mmol mmol^−1^)	pH^b^	COD balance^b^ (%)
Glucose (12.5 mM) Arabinose (12.5 mM)	0.26	0.62 ± 0.04	5.77 ± 0.1	110 ± 6
	0.13	0.54 ± 0.05	5.75 ± 0.1	94 ± 3
	0.065	0.59 ± 0.05	5.71 ± 0.1	93 ± 2
	0 (control)	0.49 ± 0.003	5.77 ± 0.1	84 ± 1

The results are the average and standard deviations for duplicate assays.

^a^mmol of hydrogen produced per mmol of sugar consumed.

^b^At the end of the batch experiment.

Concerning the hydrogen yields, higher values were also obtained for the Z/I of 0.26 and 0.065 g g^−1^ and the lowest hydrogen yield (0.49 mmol mmol^−1^ as H_2_ per substrate consumed^−1^) was observed for the control (without zeolite) (Table 1). The pH at the end of the batch experiment was similar for all the Z/I ratios and for the control, approximately 5.7.

**Figure 2:**
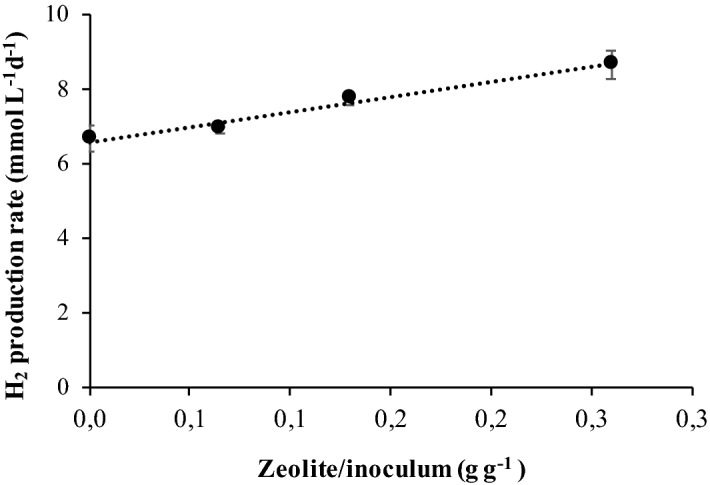
Linear correlation between Z/I ratio and the initial hydrogen production rate.

The rate of hydrogen production increased and was linearly correlated with the Z/I (Fig. 2). For the collected data it is also possible to estimate the rates of lactic, acetic and formic acid production. Apparently, there was an effect of the presence of zeolite in the decrease of lactate and formate production rates, but no significant effect was observed in the rate of acetate production (Table 2). It was also observed that for formic and lactic acid the presence of zeolite decreased the lag phase that preceded the onset of the production of these acids (about 2 days without zeolite and 1 day with zeolite for all Z/I and for both acids-not shown) (Fig. 1B,D).

**Table 2:** Estimated production rates for hydrogen, lactic, acetic and formic acid in the batch experiments with C5 and C6-sugars in the presence and absence of zeolite.

Z/I (g g^−1^)	Production rate (mmol L^−1^ day^−1^)			
	Hydrogen	Lactic acid	Acetic acid	Formic acid
0.26	8.66 ± 0.39	10.53 ± 1.23	6.07 ± 0.37	13.26 ± 0.52
0.13	7.72 ± 0.14	10.04 ± 0.87	4.11 ± 0.74	13.27 ± 0.45
0.065	6.90 ± 0.08	10.39 ± 0.89	3.60 ± 0.67	13.98 ± 0.11
0 (control)	6.66 ± 0.34	29.84 ± 0.42	3.54 ± 0.01	39.73 ± 0.47

Glucose and arabinose were totally consumed during the experiment for all Z/I ratios tested and for the control (Fig. 1A). The main end-product formed was acetic acid (reaching a maximum of 54.69 mmol L^−1^ for Z/I of 0.26 g g^−1^ (Fig. 1C). Lower amounts of lactic and formic acid were produced at similar concentrations for all the Z/I tested, and for the control (without zeolite) (Fig. 1B,D). Formic acid was produced in all the experimental conditions during the first days of operation, reaching a maximum concentration of 13 mmol L^−1^ (day 2). Afterwards, its concentration decreased to approximately 0.95 mmol L^−1^ (control Z/I of 0.26 g g^−1^) and 2.83 mmol L^−1^ Z/I of 0.13 and 0.065 g g^−1^) (day 8). This decrease was accompanied by an increase of hydrogen production and a decrease of the pH from 7 to approximately 5.7 (Table 1). The COD balance was closed in all the operational conditions with zeolite, showing that all metabolic products could be identified, except for the control (without zeolite) (Table 1).

Detailed information regarding the COD balance and the respectively soluble fermentation products formed can be found in the supplementary information (Table S1).

**Figure 3:**
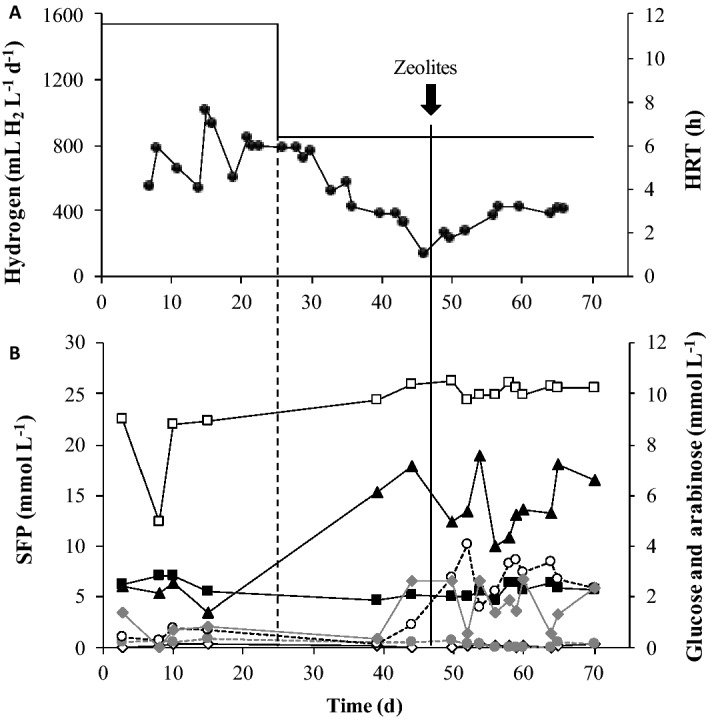
Effect of HRT and zeolite (Z/I 0.26 g g^−1^) on the bioreactor performance treating arabinose and glucose. (**A**)  Hydrogen production rate and  HRT. (**B**)  Arabinose and  glucose consumption and soluble fermentation products (SFP):  Formic acid;  Acetic acid;  Lactic acid;  n-butyrate acid;  Propionate acid.

The zeolite effect on biohydrogen production by dark fermentation was further evaluated in a continuous bioreactor (Expanded Granular Sudge Bioreactor (EGSB)) fed with C5 and C6-sugars (Fig. 3). The bioreactor operation started at a hydraulic retention time (HRT) of 12 h and at an organic loading rate (OLR) of 8.8 kg m^−3^ day^−1^ of COD. During this operational period, the maximum hydrogen production rate achieved was 1016 mL L^−1^ day^−1^ after 15 days of operation, stabilizing afterwards in approximately 800 mL L^−1^ day^−1^ around day 21 (Fig. 3A). Moreover, the maximum hydrogen yield obtained was 2.33 mmol mmol^−1^ as H_2_ per substrate consumed (day 15) (Table 3). Under these operational conditions, acetate and lactic acid were the main metabolic products and their concentrations remained stable during this operation period (approximately 6.51 and 5.34 mmol L^−1^, respectively) (Fig. 3B).

**Table 3:** Performance of the continuous bioreactor operation with C5 and C6-sugars.

HRT (h)	Z/I (g g^−1^)	Day	OLR (kg m^−3^ day^−1^)	Glucose utilization (%)	Arabinose utilization (%)	Hydrogen yield^a^ (mmol mmol^−1^)	Hydrogen production rate^b^ (mL L^−1^ day^−1^)	COD balance (%)
12	–	15	8.8	94	29	2.33	1016	71
6	–	46	17.6	81	23	0.19	143	111
6	0.26	56–66		91	18	0.53	413 ± 17^c^	103

– Not added.

^a^mmol of H_2_ per mmol of substrate consumed.

^b^Volume of H_2_ produced per litter of bioreactor volume per day.

^c^Average and standard deviation of values measured in 5 days in the indicated period.

After 25 days of operation, the HRT was decreased to 6 h and an OLR of 17.6 kg m^−3^ day^−1^ was applied. The hydrogen production rate decreased continuously and linearly over the time, reaching a minimum value of 143 mL L^−1^ day^−1^ after 46 days of operation (Fig. 3A). Hydrogen yield decreased as well to 0.19 mmol mmol^−1^ as H_2_ per substrate consumed) (Table 3). At this point, an increase in the lactic acid concentration was observed, reaching a maximum of 17.8 mmol L^−1^ (Fig. 3B).

**Figure 4:**
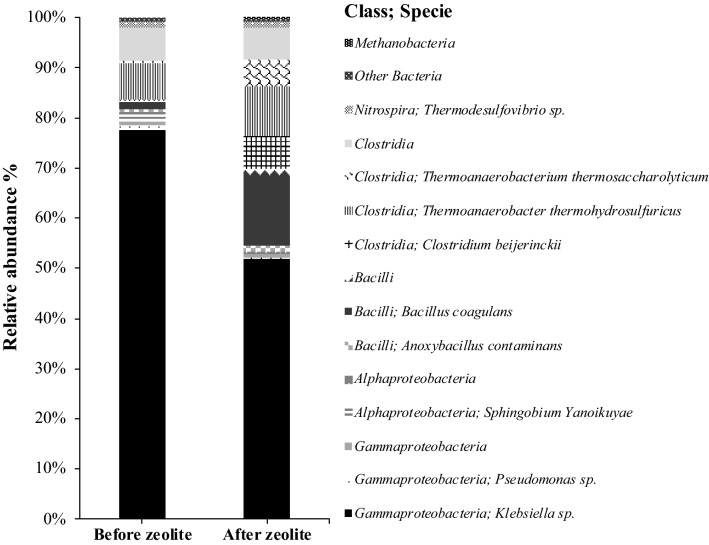
Microbial community composition in the bioreactor treating arabinose and glucose. Only the class and specie of the microorganisms with relative abundance above 1% are represented.

No methane was detected during the experiment, which is in accordance with the low percentage of methanogenic archaea observed in the microbial community (0.02% of the total microbial community, Fig. 4), meaning that inoculum pre-treatment at high temperature and low pH was efficient at inhibiting methanogens.

After the drop of the hydrogen production rate to the minimum of 143 mL L^−1^ day^−1^, zeolite type-13X (Z/I of 0.26 g g^−1^) was added to the bioreactor. This Z/I ratio of 0.26 g g^−1^ was chosen since it achieved in absolute value the higher hydrogen production in batch experiments with C5 and C6-sugars. The system performance was immediately recovered after zeolite addition (Fig. 3A) and the hydrogen production increased linearly over time during the next 8 days, reaching 413 mL L^−1^ day^−1^, which was approximately 3 times higher than the previous condition (143 mL L^−1^ day^−1^). The addition of zeolites to the continuous operation with an OLR of 17.6 kg m^−3^ day^−1^ of COD allowed to improve the hydrogen yield from 0.19 to 0.53 mmol mmol^−1^ (Table 3). It was also observed that upon zeolite addition, formic acid concentration increased from 2.64 mmol L^−1^ to approximately 7.36 mmol L^−1^ (Fig. 3B). Propionate and n-butyrate acids were also detected during the reactor’s operation, however at concentrations lower than 1 mmol L^−1^ (Fig. 3B).

The pH remained constant during the entire operation, corresponding to approximately 5.9 ± 0.7, which is in the pH range (5.0–6.0) for optimum hydrogen production by dark fermentation^18^.

Microbial community analysis showed that *Klebsiella* sp. was the most abundant microorganism along all of the operation time (Fig. 4). However, after zeolite addition its relative abundance decreased from 77.5 to 51.8%, while *Bacillus coagulans* increased its relative abundance from 1.7 to 14.1%. (Fig. 4). Moreover, organisms belonging to the clostridia class namely *Clostridium beijerinckii*, *Thermoanaerobacterium thermosaccharolyticums* and *Thermoanaerobacterium thermohydrosulfuricus* also increased its relative abundance after zeolite addition to the bioreactor from 0.33 to 6.6%, 0.44 to 5.4% and 7.2 to 9.9%, respectively. Other microorganisms were also detected in the microbial community but in very low relative abundance (< 1%).

Glucose was detected in residual amounts while arabinose concentrations were maintained stable (around 10 mmol L^−1^) all along the operation time (Fig. 3B). The COD balance was closed during the bioreactor operation with an HRT of 6 h before and after zeolite addition, showing that all metabolic products could be identified, but not during the continuous operation with an HRT of 12 h (Table 3). Detailed information regarding the COD balance and the respectively soluble fermentation products formed can be found in the supplementary information (Table S2).

### Zeolite effect in biohydrogen production from *Sargassum* sp

**Figure 5:**
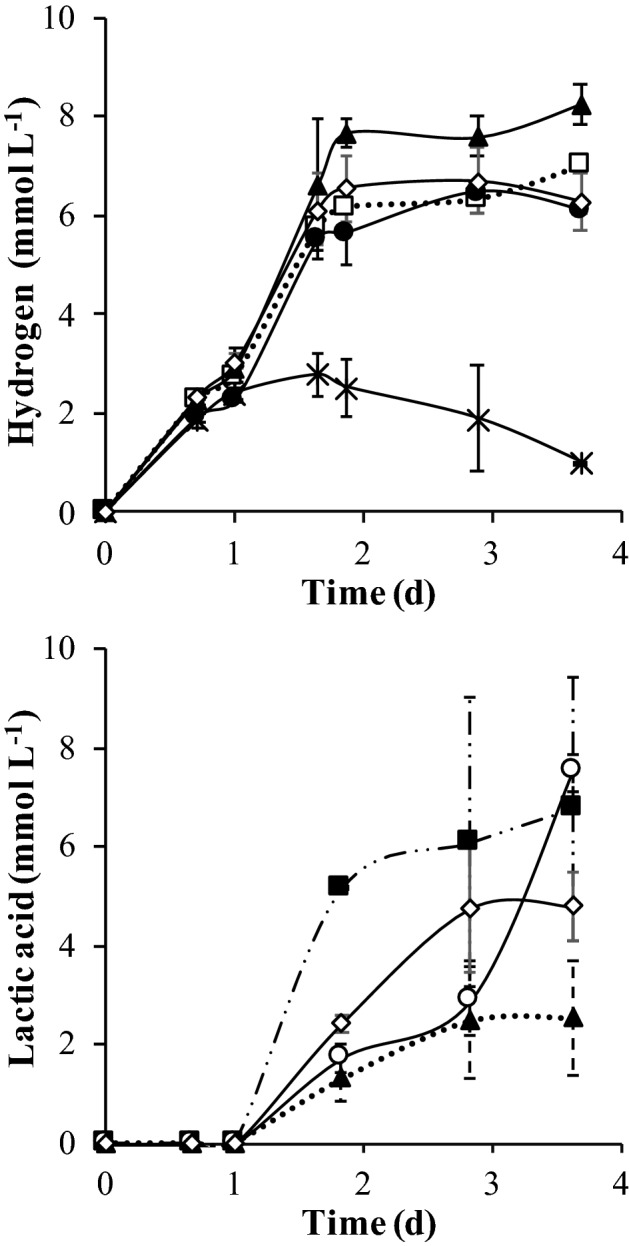
Cumulative hydrogen production for the Z/I ratio of  0.26 g g^−1^,  0.13 g g^−1^,  0.065 g g^−1^, and for the  control, and respectively lactic acid production for the Z/I ratio of  0.26 g g^−1^;  0.13 g g^−1^,  0.065 g g^−1^ and for the  control.  Blank assay without zeolite and *Sargassum* sp. The results are the average and standard deviations for duplicate assays.

**Figure 6:**
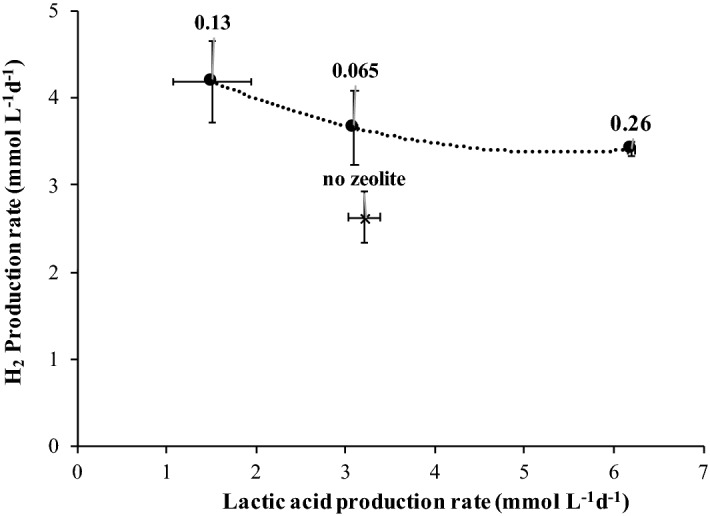
Rates of hydrogen and lactate production for the experiments ● with and Χ without zeolite. Numbers represent the Z/I ratio.

Hydrogen production by dark fermentation of *Sargassum* sp. biomass was also improved in the presence of zeolite type-13X (Fig. 5). The highest hydrogen production was obtained for the Z/I of 0.13 g g^−1^ (8.28 mmol L^−1^), which was approximately 27% higher than the control (6.10 mmol L^−1^) (Fig. 5). The Z/I of 0.26 g g^−1^ achieved a hydrogen production of 7.03 mmol L^−1^. Concerning the rates of hydrogen and lactic acid production (Table 4, Fig. 6) they were inversely related in the presence of zeolites, but no pattern was observed for the influence of the Z/I in the H_2_ production rate, with the maximum rate occurring for the intermediate Z/I of 0.13 g g^−1^.

**Table 4:** Hydrogen and lactic acid production rate (mmol L^−1^ day^−1^) in batch experiments with *Sargassum* sp. biomass in the presence and absence of zeolite.

Z/I (g g^−1^)	Production rate (mmol L^−1^ day^−1^)	
	Hydrogen	Lactic acid
0.26	3.41 ± 0.08	6.20 ± 0.04
0.13	4.19 ± 0.47	1.51 ± 0.44
0.065	3.66 ± 0.43	3.10 ± 0.03
0 (control)	3.21 ± 0.30	2.63 ± 0.18

No methane was detected during the assays in all of the conditions tested. In the blank assay, containing only anaerobic sludge, hydrogen was produced (2.79 mmol L^−1^), which means that part of the hydrogen produced, in the assays containing *Sargassum* sp., may be derived from the residual substrate associated with the inoculum sludge (Fig. 5).

Up to 13 mmol L^−1^ of acetic, iso-butyric, n-butyric, formic and propionic acid were formed (Figure S1). The COD balance was closed for the control and the Z/I of 0.26 g g^−1^ showing that all metabolic products could be identified, but not for the Z/I of 0.13 g g^−1^ (Table 5).

Detailed information regarding the COD balance and the soluble fermentation products can be found in the supplementary information (Table S3). The pH during the experiment decreased from 7 to approximately 6.6 in all operational conditions tested (Table 5).

**Table 5:** Hydrogen yields, pH and COD balance from batch experiments with *Sargassum* sp. biomass.

Substrate	Z/I (g g^−1^)	Hydrogen yield^a^ (mmol g^−1^)	pH^b^	COD balance^b^ (%)
*Sargassum* sp.	0.26	2.41 ± 0.04	6.60 ± 0.02	99 ± 9
	0.13	2.98 ± 0.28	6.59 ± 0.03	81 ± 8
	0.065	2.56 ± 0.23	6.60 ± 0.01	90 ± 1
	0 (control)	2.13 ± 0.03	6.56 ± 0.04	95 ± 4

The results are the average and standard deviations for duplicates.

^a^ expressed as mmol H_2_ per g of *Sargassum* sp. (in VS).

^b^ at the end of batch experiment assays.

## Discussion

The addition of a zeolite Type 13X improved the yields and the rates of biohydrogen production by dark fermentation from simple carbohydrates (C5 and C6-sugars) and from a complex waste *Sargassum* sp. biomass.

For the experiments with sugars a linear correlation was observed between the H_2_ production rate and the Z/I ratio (Fig. 2). The production of other fermentative end-products rather than acetic acid by mixed-cultures can lead to low hydrogen yields comparing with the theoretical ones^19^. This was the case of the batch experiments performed with C5 and C6-sugars, were lactic and formic acid were produced besides acetic acid.

During high-rate dark fermentation systems operation, the decrease of the HRT, and thus increase of applied OLR, can often cause instability on the bioreactor performance, leading to lower hydrogen yields^20^. In the present study, the decrease of the HRT from 12 h (OLR of 8.8 kg m^−3^ day^−1^ of COD) to 6 h (ORL of 17.6 kg m^−3^ day^−1^ of COD) turned the performance of continuous C5 and C6-sugars fermentation unstable and led to a decrease on hydrogen production rate from approximately 800–143 mL L^−1^ day^−1^. Glucose was preferentially consumed compared with arabinose. The presence of a rapidly biodegradable source of carbon, as glucose, can inhibit the synthesis of enzymes involved in the metabolism of other carbon sources, leading to the repression of arabinose catabolism and, thus, to a preferential uptake of glucose instead of arabinose^20,21^.

The hydrogen production rates observed were lower than the ones reported in similar studies (Table 6). For instance, Abreu et al.^20^ reported a hydrogen production rate of 2700 mL L^−1^ day^−1^ at an HRT of 8 h in an extreme thermophilic EGSB reactor fed with C5 and C6-sugars (at 5 g COD L^−1^). In the same study, the hydrogen yield obtained at steady state was 0.77 mmol mmol^−1^ as H_2_ per substrate consumed^−1^ (HRT 8 h and fed concentration of 5 g COD L^−1^), which was 4 times higher than the obtained in this work (0.19 mmol mmol^−1^) (HRT 6 h and fed concentration of 4.4 g COD L^−1^)^20^ (Table 6). Nevertheless, in this study zeolite addition to the bioreactor has shown to be an interesting strategy to counteract the decrease on hydrogen yields caused by the increase of the applied OLR. A remarkable recovery of the system performance operating at high ORL (17.6 kg COD m^−3^ day^−1^), with a threefold improvement in hydrogen production, from 143 to 413 mL L^−1^ day^−1^ (Fig. 3A), as well as an increment in the hydrogen yield from 0.19 to 0.53 mmol mmol^−1^ (Table 3) was observed.

**Table 6:** Comparison of hydrogen yields obtained in this study in continuous and batch mode with the ones reported in literature.

Process	Operation mode	HRT (h)	Inoculum	Substrate	Zeolite	Hydrogen yield^a^ (mmol mmol^−1^)	References
Dark fermentation	Continuous	12	Granular sludge	Glucose and arabinose (12.5 + 12.5 mM)	**–**	2.33	This study
		6			**–**	0.19	
		6			Zeolite type 13X	0.53	
	Batch	**–**	Granular sludge	Glucose and arabinose (12.5 + 12.5 mM)	**–**	0.53 ± 0.02	This study
		**–**			Zeolite type 13X	0.70 ± 0.03	
		**–**	Granular sludge	*Sargassum* sp.	**–**	2.63 ± 0.03^b^	
		**–**			Zeolite type 13X	3.37 ± 0.16^b^	
	Continuous	8	Heat treated sludge granules	Glucose and arabinose (12.5 + 12.5 mM)	**–**	0.77 ± 0.05	^20^
		10	Granular sludge	Glucose (13.8 mM)	**–**	0.80 ± 0.03	^39^
		10	Granular sludge	Arabinose (33.3 mM)	**–**	1.10 ± 0.01	^39^
Dark fermentation and AD	Batch	**–**	*Caldicellulosiruptor saccharolyticus*	*Sargassum* sp.	**–**	91.30 ± 3.30^c^	^3^
Dark- and photo-fermentation	Batch	**–**	Heat-treated anaerobic sludge	*Arthrospira platensis* wet biomass	Natural zeolite	337^d^	^13^
		**–**	*Lactobacillus delbrueckii* NBRC13953 and *R. sphaeroides* RV	Glucose	**–**	7.10	^40^

^a^mmole of H_2_ per mmole of substrate.

^b^H_2_ produced per mass of *Sargassum* sp. (in VS) (mmol g^−1^).

^c^Volume of H_2_ produced per mass of *Sargassum* sp. (in VS) (L kg^−1^).

^d^Volume of H_2_ produced per mass of cellular dry weight (in VS) (L kg^−1^).

^e^mmol of H_2_ produced per bioreactor culture volume per hour of operation.

The metabolic profile obtained in the batch and continuous bioreactors, as well as the microbial community composition results, shows that several metabolic pathways were taking place simultaneously and were influenced by the presence of zeolites. In dark fermentation, hydrogen can be produced by two different pathways, i.e., mixed acid fermentation (or formic acid fermentation) and the clostridial-type fermentation (or butyric acid fermentation)^22^. Mixed acid fermentation is characteristic of bacteria that belong to the *Enterobacteriaceae* family, such as *Klebsiella* sp., while clostridial-type fermentation is typical in bacteria belonging to the *Clostridium* and *Bacillus* genera^22^. For both fermentation types, glycolysis is the first step in which glucose is converted into pyruvate with the formation of nicotinamide adenine dinucleotide (NADH)^22^. Depending on the fermentation type, different metabolic profiles can be obtained from pyruvate. In mixed acid fermentation the end products include lactate, formate, acetate, succinate, ethanol and the gases H_2_ and CO_2_^22^. In butyric acid fermentation, butyrate can be produced along with acetate, lactate, ethanol and H_2_ and CO_2_^23^. In both types of fermentation, acetate can be produced from acetyl-CoA in order to maximize ATP production, and to re-oxidize the NADH in order to provide NAD^+^ for the glycolytic pathway^1^. The accumulation of formic acid in batch experiments, during the first days of incubation, suggest that bacteria belonging to the *Enterobacteriaceae* family (known to perform the mixed-acid fermentation^22^) were the most active in this period (Fig. 1D). In this pathway, the pyruvate from glycolysis is cleaved by the enzyme pyruvate:formate lyase (Pfl) to form formic acid and acetyl-CoA^22^. Then, acetyl-CoA can be converted in acetate while formic acid can be degraded into hydrogen and carbon dioxide by formate:hydrogen lyase (FHL) that is activated at low pH^1,22^. Indeed, in the batch experiments, formic acid reached residual concentrations (below 2.8 mmol L^−1^), which was accompanied by the production of hydrogen and the decrease of the pH to 5.7.

A similar outcome was observed in the continuous experiment (Fig. 3). The production of acetic and formic acid during the process suggests that mixed-acid fermentation was the main metabolic pathway occurring. Analysis of the bioreactor microbial community showed the presence of potential hydrogen producing bacteria belonging to *Klebsiella*, *Bacillus*, *Clostridium* and *Thermoanaerobacterium* genera. Indeed, *Klebsiella* sp., that belongs to the *Enterobacteriaceae* family, was the most abundant microorganism in the continuous bioreactor operation before and after zeolite addition, although its relative abundance decreased from 77.5 to 51.8% (Fig. 4). Several studies reported the potential use of *Klebsiella* sp. to produce hydrogen under anaerobic conditions from a variety of carbohydrates and from more complex residues such as corn stalk hydrolysate^20,24,25^.

Moreover, *Klebsiella* sp. such as *Klebsiella pneumoniae* have been extensively studied due to its ability to produce several value-added products namely, lactic acid and ethanol under anoxic conditions^25^.

The presence of butyrate in the batch and continuous experiments can also be indicative of the performance of the clostridial-type fermentation, where the end products are hydrogen and acetate or butyrate, although relatively low butyrate concentrations could be detected (lower than 1 mmol L^−1^)^23^. Indeed, *Clostridium, Thermoanaerobacterium* and *Bacillus* genera, known to perform the clostridial-type fermentation were identified in the continuous bioreactor microbial community and account for 0.33%, 0.44% and 1.7% of the total microbial community before zeolite addition and 6.6%, 5.4% and 14.1% after zeolite addition (Fig. 4). Several species belonging to *Thermoanaerobacterium* genera namely *T. thermosaccharolyticum* were reported in literature to be promising candidates in lignocellulosic biomass conversion to biohydrogen with the simultaneous production of acetic and butyric acid as the principal fermentative end-products^26,27^. The analysis of the bioreactor microbial community showed the presence of microorganisms close related to *B. coagulans* and *C. beijerinckii*. These bacteria are capable of hydrogen production from a variety of carbon sources (e.g. l-arabinose, d-glucose, d-fructose, sucrose, maltose, starch and others)^28^. Masset et al.^29^ showed *C. beijerinckii* ability to produce hydrogen from formic acid. The authors pointed out that the ability of this bacteria to re-consume formate suggests the existence of a new metabolic pathway in clostridia^29^. Although, *Bacillus* genus is known to be hydrogen producer, some *Bacillus* species namely *B. coagulans* are also capable of producing lactic acid. Bischoff et al.^30^ isolated from a composted dairy manure a *B. coagulans* strain capable of converting mixed sugars of glucose, xylose and arabinose to l-lactic acid with 85% yield at a temperature of 50 °C. Indeed, lactic acid was detected in both batch and continuous experiments at considerable concentrations. The deviation of the metabolism for lactic acid production is associated with a decrease in hydrogen yields, since it is produced by a zero-hydrogen balance pathway^16,20^. In fact, the sharp increase in lactate concentration observed after lowering the HRT from 12 to 6 h in the continuous bioreactor was accompanied by a severe decrease in hydrogen production. The increase of lactic acid concentration with the decrease of the HRT and decrease on hydrogen production was already reported in EGSB reactors treating arabinose and glucose^20^. With the organic load increase there was, likely, an excess of reducing power overloading the H_2_ producing pathways and, thus, lactate was produced as a way to cope with the excess of protons generated. The addition of zeolite triggered a fluctuation in the production of lactate and formic acid leading to an increase in hydrogen production (Fig. 3A).

The link between hydrogen and lactate production was evident also in the batch assay with *Sargassum* sp. biomass. The rates of hydrogen and lactic acid production were inversely related in the presence of zeolites (Fig. 6), but no pattern was observed for the influence of the Z/I ratio in the H_2_ production rate, as observed for the sugars experiment. A maximum rate was observed for the intermediate Z/I of 0.13 g g^−1^ (Table 4). The H_2_ production rates were within a narrow range, but there is a clear indication that zeolite influenced in a higher extent the rate of lactate production.

Batch experiments performed with *Sargassum* sp. biomass showed that this residue can be efficiently used by mixed-cultures for hydrogen production via dark fermentation. The presence of zeolite improved hydrogen production from 6.10 mmol L^−1^ (control without zeolite) to approximately 8.3 mmol L^−1^ for Z/I of 0.13 g g^−1^ demonstrating the zeolite potential to enhance hydrogen production from lignocellulosic biomass. It was previously reported that *Sargassum* sp. biomass could be a suitable substrate for the generation of hydrogen enriched biogas (10–25% of H_2_) in a two-step system, combining dark fermentation from pure culture and AD process (Table 6)^3^. Therefore, zeolite addition to this two-step system can be a suitable strategy to enhance hydrogen production, increasing thereby the energy potential of the biogas produced from *Sargassum* sp. biomass.

Zeolites were used before in biological processes, such as AD and in a two-step process that coupled dark- and photo-fermentation in order to improve methane and hydrogen production, respectively^8,13,14^ (Table 6). In most of the studies, zeolite effect was attributed to the removal of NH_4_^+^ ions from the fermentation effluent due to its ion-exchanger capacity^8,13,14^. In this study, ammonium was not present in the fermentation medium and biohydrogen production was improved by adding zeolite in both batch and continuous experiments. Therefore, in this case, zeolites effect can be related to its ion-exchanger capacity or to its ability to adsorb molecules other than ammonium. In fact, besides NH_4_^+^, zeolites can adsorb different types of molecules. Specifically, zeolite type-13X was described as capable to adsorb CO_2_, CH_4_ and/or CO for hydrogen purification from gaseous mixtures^31^. In addition, other functions may be attributed to zeolites which can contribute to the observed improvement on hydrogen production, such as the presence of different catalytic sites (Brönsted and Lewis sites) in zeolites structure and their ability to function as a sink of trace elements which are important to provide optimal growth conditions to hydrogen producing microorganisms. Indeed, a previous study concluded that zeolites improved the biogas process efficiency, not only by reducing the ammonia concentration in the media, but also because its porous structure could function as a supplement of trace elements (i.e., Ca, Na, K and Ba), thus improving the activity of methanogens^8,32^. Another important feature of zeolites is the high-capacity to function as a matrix for the immobilization of microorganisms^7,33^. This was not likely the case in the present study, since the inoculum consisted of granular sludge where microorganisms are already self-immobilized.

The adsorption capacity of zeolite can be extended to protons. Since the dark fermentation process occurs at pH range between 5.0 and 6.0, protons in solution are most likely adsorbed through zeolite by ion-exchange. In this study it was hypothesized that these protons sink may reduce the NADH/NAD^+^ redox imbalance that is usually associated to formation of more reduced products such as lactate. In fact, it is worth to notice that, during the continuous operation, zeolite addition triggered a dynamic fluctuation between formate (a precursor of H_2_ production) and lactate, conducting ultimately to an increase in H_2_ production (Fig. 3A). The multitask potential of zeolites in several biotechnological processes suggests that its effect on dark fermentation may be the result of not one but several properties.

The improvement on the biohydrogen production can be linked to metabolic changes induced by zeolites as they affected the microbial community structure. The metabolic profile obtained in the batch and continuous bioreactors show that several metabolic pathways were taking place simultaneously and were influenced by the presence of zeolite, favoring hydrogen production.

Dark fermentation is a complex process that still has a long way to become a competitive technology for hydrogen production. The most suitable operational conditions still need to be optimized, since this fermentative process is influenced by several operational parameters such as inoculum source, temperature, pH, HRT, H_2_ partial pressure and fermentative end-products^34^. The obtained results demonstrate zeolite potential to improve thermophilic biohydrogen production from C5 and C6-sugars and from lignocellulosic wastes such as *Sargassum* sp. biomass, showing the versatility and suitability of this material, and opening a wide range of possibilities to be incorporated in biological processes for hydrogen production from renewable feedstocks.

## Material and Methods

### Batch experiments

Batch experiments were conducted in 120 mL serum bottles with a working volume of 50 mL, containing anaerobic medium with 0.6 mL g^−1^ of Chemical Oxygen Demand (COD) of macronutrients^20^, 10 mmol L^−1^ of sodium bicarbonate, resazurin at a final concentration of 0.5 mg L^−1^ and 15 mmol L^−1^ of sodium 2-bromoethanesulfonate (BES) to inhibit the methanogenic activity. Anaerobic granular sludge (with a final concentration of 7.72 g L^−1^ of volatile solids (VS)), collected from a brewery industry (Lisbon, Portugal), was used as inoculum and a mixture of l-arabinose and glucose monohydrated (25 mmol L^−1^, 1:1) as substrate. Three different amounts of zeolite type-13X to inoculum (in VS) ratios were tested: 0.26, 0.13 and 0.065 g g^−1^. The bottles were sealed and the headspace flushed with N_2_/CO_2_ (80/20%) to guarantee the anaerobic conditions and the buffer effect. Sodium sulfide (0.8 mmol L^−1^) was used as a reducing agent to eliminate the residual O_2_ concentrations in the medium. The assays were performed at 70 °C, 150 rpm and at an initial pH of 7.0. A schematic representation of the batch experiment is described in figure S2.

The zeolite used consisted in the commercial type-13X, known as molecular sieves and was obtained from Sigma Aldrich. It is composed by 1 Na_2_O:1 Al_2_O_3_:2.8 ± 0.2 SiO_2_:xH_2_O, the spherical bleads of the zeolite have a size of 4–8 mesh with an average pore diameter of 10 Å and are composed essentially by the *faujasite* structure^35^. This type of structure is composed by 3-dimensional channel system with supercages and 12-ring pore openings, with high surface area, low Si/Al ratio which confer higher cation exchange capacity and high thermal and chemical stability. In addition, the low Si/Al ratio, saturated in aluminium, offers excellent properties in terms of adsorption capacity^36^.

Consumption of arabinose and glucose and production of hydrogen and other soluble fermentation products (SFP) were monitored during the experiments.

A different set of batch assays was performed using *Sargassum* sp. as substrate, in the same conditions described for the batch assays with glucose and arabinose, with exception of the working volume (serum bottles and working volume of 160 and 60 mL, respectively).

*Sargassum* sp. was collected during spring from a location in the north coastline of Portugal (Póvoa de Varzim). The macroalgae was dried at room temperature and then milled into pieces with less than 0.5 cm^3^. The *Sargassum* sp. characterization can be found in^3^. To increase the soluble COD, the macroalgae was autoclaved at 121 °C and 1 bar for 10 min^3^.

In this experiment, a ratio of 1.54 g of inoculum (in VS) per g of *Sargassum* sp. (in VS) was used and the ratios 0.26 and 0.13 g zeolite per g of inoculum (in VS) were tested.

For both experiments a blank assay without inoculum and a control without zeolite were included. In the assay with *Sargassum* sp. an additional blank assay with only anaerobic granular sludge without any substrate was also performed for quantification of the hydrogen produced from the residual substrate present in the inoculum. For each condition, four replicates were performed. To avoid hydrogen leaks, gas composition was determined in two of the bottles and liquid samples were collected from the remaining two bottles for VFA, lactic acid and sugars quantification. pH was monitored in both experiments.

**Figure 7:**
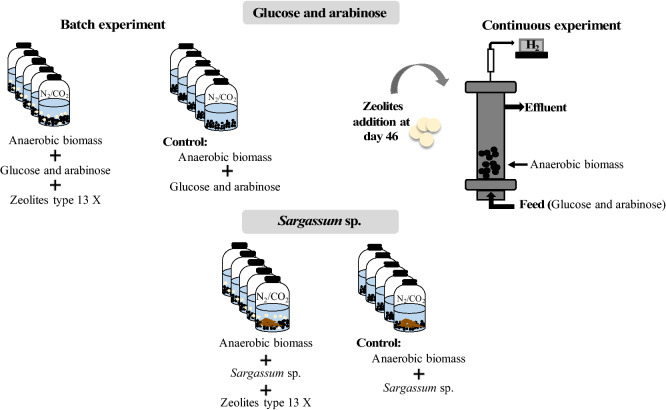
Schematic representation of the batch and continuous experiments for biohydrogen production by dark fermentation.

A schematic representation of the batch experiments for biohydrogen production by dark fermentation is shown in Fig. 7.

### Continuous bioreactors operation

Anaerobic granular sludge from a brewery industry (Lisbon, Portugal) was previously acclimated for 2 months on an expanded granular sludge bed bioreactor (EGSB) at extreme thermophilic conditions (70 °C) and pH 5.5, to select for hydrogen producers and to inhibit methanogens. This sludge (0.08 ± 0.01 g VS g^−1^) was used as inoculum (300 mL) in a stainless steel EGSB reactor (internal diameter of 5.65 cm and total volume of 2.68 L) with a working volume of 2.14 L. The reactor was fed with l-arabinose and glucose monohydrated [1:1 (w/w)] at a concentration of 4.4 g L^−1^ (in COD) and operated in continuous mode at 70 ± 2 °C during 71 days. The feed was supplemented with 0.6 mL g^−1^ COD of macronutrients and sodium bicarbonate (NaHCO_3_) at a concentration of 2 g L^−1^ for pH control; and it was maintained at 4 °C to minimize acidification, as described elsewhere^20^. The pH was controlled by the addition of sodium bicarbonate (NaHCO_3_) to the bioreactor feed. Afterwards, the pH was monitored throughout the continuous operation (approximately 5.9 ± 0.7). The bioreactor was operated in the first 25 days with a hydraulic retention time (HRT) of 12 h and with an organic loading rate (OLR) of 8.8 kg m^−3^ day^−1^ of COD. Thereafter the HRT was decreased to 6 h during 45 days. At this operation period an OLR of 17.6 kg m^−3^ day^−1^ of COD was applied to the bioreactor. Zeolite type-13X was added to the bioreactor (in a ratio of 0.26 g of zeolite per g of inoculum (in VS)) when the hydrogen production dropped after 46 days of operation. A schematic representation of the continuous experiment is described in Fig. 7.

Gas samples were collected for hydrogen and methane measurements. Volatile fatty acids (VFA), lactic acid, glucose and arabinose concentrations were determined in liquid samples collected from the effluent.

Sludge samples (approximately 0.5 mL) were collected for microbial community taxonomic characterization at day 46 (immediately before zeolite addition) and at day 66 (after zeolite addition). Samples were centrifuged, washed with PBS and stored at − 20 °C. Total genomic DNA was extracted by using the FastDNA SPIN kit for soil (MP Biomedicals LLC, Santa Ana, CA). 16S rRNA genes were sequenced by Illumina MiSeq, using the universal prokaryotic primer pair 515-f/806-r^37^. Sequencing and data analysis were performed by RTL (Research testing Laboratory, Texas, US) as described elsewhere^38^. Sequencing reads were submitted to the European Nucleotide Archive (ENA) under the study accession number PRJEB23747 (sample SAMEA104420766, before zeolite addition; sample SAMEA104420767, after zeolite addition).

### Analytical methods

Hydrogen and methane concentration in batch and continuous experiments were analyzed by gas chromatography using a BRUKER SCION 456 with a column molsieve (MS-13 × 80/100 mesh) and connected to a thermal conductivity detector. Argon was used as carrier gas at a rate of 30 mL min^−1^ and the injector, detector and column temperatures were set at 100, 130, and 35 °C, respectively. VFA, lactic acid and sugars (i.e., arabinose, glucose) were determined by high performance liquid chromatography using a chromatograph (Jasco, Japan) with an Aminex column (HPX-87H Ion 300 mm × 7.8 mm^2^); Sulfuric acid (0.01 N) at a flow rate of 0.7 mL min^−1^ was used as mobile phase. Column temperature was set at 60 °C and was used a retention time of 50 min. Detection of VFA’s and lactic acid was made through a UV detector at 210 nm, and the detection of glucose and arabinose was made by using an RI detector. pH was measured by using a benchtop metre InoLabVR pH 7110 (WTW, Weilheim, Germany). Determination of COD was performed using the commercial kits LCK 014 (colorimetric method) from Hach Lange, Düsseldorf, Germany, following the manufacturer’s instructions.

### Statistical analysis

Significant differences between biological samples, collected from batch incubations with and without zeolite, were determined by applying a t test using Microsoft Excel (unequal variance t test was applied to batch experiments with *Sargassum* sp. and equal variance t test to the batch experiments with sugars, as determined by the results of the variance analysis obtained with the F test). The significant threshold was set at *P* = 0.05.

## Supplementary Information

Supplementary Information.

## Abbreviations

ATP: Adenosine triphosphate
AD: Anaerobic digestion
BES: Sodium 2-bromoethanesulfonate
COD: Chemical oxygen demand
DNA: Deoxyribonucleic acid
EGSB: Expanded granular sludge bed bioreactor
HRT: Hydraulic retention time
OLR: Organic loading rate
NADH: Nicotinamide adenine dinucleotide
SFP: Soluble fermentation products
VFA: Volatile fatty acids
VS: Volatile solids
Z/I: Zeolite/inoculum ratio
