# Supplementary material for: Zeolite addition to improve biohydrogen production from dark fermentation of C5/C6-sugars and Sargassum sp. biomass
Source: Sci Rep. 2021 Aug 11;11:16350. doi: 10.1038/s41598-021-95615-1 (PMC8358045; doi:10.1038/s41598-021-95615-1)
Supplement: Supplementary file 1 — Supplementary Information. [file 41598_2021_95615_MOESM1_ESM.docx]

**Supplementary material**

**Zeolite addition to improve biohydrogen production from dark fermentation of C5/C6-sugars and *Sargassum* sp. biomass**

Silva ARM^1a^, Abreu AA^1‡b^, Salvador AF^1c^, Alves MM^1d^, Neves IC,^1,2,f^ Pereira MA^1e^*

^1^Centre of Biological Engineering, University of Minho, 4710-057 Braga, Portugal.

^2^CQUM-Centre of Chemistry, University of Minho, 4710-057 Braga, Portugal

[^a^rita.silva@ceb.uminho.pt](mailto:arita.silva@ceb.uminho.pt), ^b^abreu.angela@gmail.com, [^c^asalvador@ceb.uminho.pt](mailto:casalvador@ceb.uminho.pt), [^d^madalena.alves@deb.uminho.pt](mailto:dmadalena.alves@deb.uminho.pt), ^e^ineves@quimica.uminho.pt, [^f^alcina@deb.uminho.pt](mailto:falcina@deb.uminho.pt).

**Figure S1.** Metabolic profile of the batch experiment performed with *Sargassum* sp*.* biomass. The results are the average and standard deviations for triplicates assays.

**Table S1.** COD balance and soluble fermentation products as % of the total COD obtained in batch assays with C5 and C6-sugars and *Sargassum* sp. biomass as substrates.

| **Substrate** | **Z/I** (g g^-1^) | **Soluble fermentation products (SFP)**  (% of total COD) | | | | | | **Total COD ^(a)^**  (g L^-1^) | **COD balance ^(b)^ ^(%)^** |
| --- | --- | --- | --- | --- | --- | --- | --- | --- | --- |
|  |  | **Lactic acid** | **Acetic acid** | **Formic acid** | **n-Butyric acid** | **iso-Butyric acid** | **Propionic acid** |  |  |
| **Glucose and arabinose**  **(4.4 g COD L^-1^)** | 0.26 | 33.6 ± 0.3 | 67.6 ± 0 | 0.76 ± 0 | n.d | n.d | n.d | 4.9 ± 0.3 | 110 ± 6 |
|  | 0.13 | 31.3 ± 0.3 | 68.4 ± 0.2 | 0.27 ± 0.02 |  |  |  | 4.1 ± 0.1 | 94 ± 3 |
|  | 0.065 | 32.9 ± 0 | 67.7 ± 0.1 | 0.38 ± 0.02 |  |  |  | 4.1 ± 0.1 | 93 ± 2 |
|  | control | 31.5 ± 0 | 67.5 ± 0 | 0.99± 0 |  |  |  | 3.7 ± 0 | 84 ± 1 |
| ***Sargassum* sp. (2.72 g COD L^-1^)** | 0.26 | 23.3 ± 0.3 | 43.5 ± 0.2 | 0.86 ± 0 | 7.3 ± 0 | 9.3± 0 | 15.7 ± 0.1 | 2.7 ± 0.5 | 99 ± 9 |
|  | 0.13 | 15.1 ± 0.2 | 48.6 ± 0.2 | 0.82 ± 0 | 5.9± 0.1 | 11.3 ± 0.1 | 18.2 ± 0.1 | 2.2 ± 0.5 | 81 ± 8 |
|  | 0.065 | 24 ± 0.1 | 39.0 ± 0.1 | 0.50 ± 0 | 39.0± 0 | 6.0 ± 0 | 8.0 ± 0 | 2.5 ± 0 | 90 ± 1 |
|  | control | 28 ± 0 | 38.2 ± 0.1 | 0.86 ± 0 | 8.3 ± 0 | 9.8 ± 0 | 14.6 ± 0.1 | 2.6 ± 0.1 | 95 ± 1 |

n.d - not detected

^(a)^ Total COD regarding the soluble fermentation products produced
^(b)^ COD balance at the end of batch experiments.

**Table S2.** COD balance and soluble fermentation products as % of total COD obtained in the continuous bioreactor operation fed with C5 to C6-sugars.

| **HRT** (h) | **Z/I**  **(g g^-1^)** | **Day** | **Feed**  **concentration**  (g COD L^-1^) | **Soluble fermentation products (SFP)**  (% of total COD) | | | | | **Glucose remaining** | **Arabinose remaining** | **Total COD ^a^**  (g L^-1^) |
| --- | --- | --- | --- | --- | --- | --- | --- | --- | --- | --- | --- |
|  |  |  |  | **Lactic acid** | **Acetic acid** | **Formic acid** | **n-Butyric acid** | **Propionic acid** |  |  |  |
|  |  |  |  |  |  |  |  |  | (% of total COD) | |  |
| **12** | - | 15 | 4.4 | 14.6 | 1.1 | 14.7 | 6.4 | 1.8 | 3.5 | 59.0 | 2.41 |
| **6** | - | 44 |  | 40.4 | 0.9 | 7.9 | 2.4 | 0 | 11.9 | 36.6 | 4.22 |
| **6** | 0.26 | 65 |  | 42.1 | 2.4 | 9.1 | 2.5 | 0.7 | 3.1 | 40.0 | 4.11 |

^(a)^ Total COD regarding the soluble fermentation products produced and the remaining glucose and arabinose concentrations.

- Not added.
